# A comprehensive study of the potential phytomedicinal use and toxicity of invasive *Tithonia* species in South Africa

**DOI:** 10.1186/s12906-018-2336-0

**Published:** 2018-10-03

**Authors:** Aitebiremen Gift Omokhua, Muna Ali Abdalla, Johannes Van Staden, Lyndy Joy McGaw

**Affiliations:** 1 Phytomedicine Programme, Department of Paraclinical Sciences, University of Pretoria, Private Bag X04, Onderstepoort, 0110 South Africa; 2 Research Centre for Plant Growth and Development, School of Life Sciences, University of KwaZulu-Natal, Private Bag X01, Scottsville, 3201 South Africa; 3 Deparment of Food Science and Technology, Faculty of Agriculture, University of Khartoum, 13314 Khartoum North, Sudan

**Keywords:** Alien, Invasive, Phytochemical, Antimicrobial, Selectivity index, Toxicity, Genotoxicity, South Africa

## Abstract

**Background:**

*Tithonia diversifolia* and *T. rotundifolia* belong to the Asteraceae family and are native to Mexico and Central America. These plants have become invasive in parts of tropical Africa and Asia where they have become an ecological, agricultural and economic burden. *Tithonia diversifolia* is exploited by locals in its native and most parts of its invasive range as a source of medicines; however, *T. rotundifolia* is only used for medicinal purposes in one country in the native range (Venezuela) and none in the invasive range. Although *T. diversifolia* has been studied for different biological activities, little or no attention has been given to *T. rotundifolia*. This study compared the antimicrobial activity, phytochemistry, identification of bioactive compound(s) and toxicity levels of different leaf extracts and fractions of *T. diversifolia* and *T. rotundifolia*.

**Methods:**

Antimicrobial activity was evaluated against seven pathogenic bacteria, four non-pathogenic *Mycobacterium* species and three fungal species using serial microdilution assays. Phytochemical contents were determined through standard methods of analysis. UPLC/MS was used to analyse the fractions to identify possible bioactive compounds that may be responsible for bioactivity, while toxicity tests were carried out using the colorimetric MTT assay and the Ames test.

**Results:**

Both species had a range of antimicrobial activity against bacterial, mycobacterial and fungal species. However, *T. rotundifolia* displayed better activity against most of the strains tested with minimum inhibitory concentration values ranging between 0.01 and 0.07 mg/ml. Both species were rich in phenolics, flavonoids and tannins. Tagitinin A was identified as the main compound present in both species, and this compound may be responsible for the antimicrobial activity displayed. Toxicity tests showed that *T. diversifolia* was cytotoxic at concentrations used in this study, while *T. rotundifolia* was not. Both species did not show any mutagenic/genotoxic effects.

**Conclusion:**

The above results suggest that both species may be further developed as a source of antimicrobials for the treatment of infections caused by opportunistic pathogens. They may also serve as alternatives to highly exploited plant species with the same medicinal properties. However, *T. diversifolia* should be used with caution as it may be toxic.

## Background

The genus *Tithonia,* belonging to the family Asteraceae, comprises eleven species, namely *Tithonia excelsa*, *T*. *playlepsis*, *T. fruticosa*, *T*. *tagitiflora*, *T. speciosa*, *T*. *scaberrima*, *T. glaberrima*, *T. ovata*, *T*. *tubiformis*, *T. diversifolia* and *T. rotundifolia*. These species are of Mexican, Central American and Cuban origin [1–3]. However, species such as *T. diversifolia* and *T. rotundifolia* are now found in most parts of the world including Asia and Africa where they have become invasive [4–7]. The two species were introduced as ornamental plants into South Africa in the early 1900s [5]. *Tithonia diversifolia,* which is the most common in West Africa, was thought to have been introduced into the region through *Zea mays* importation from Israel in the late 1970s [8]. Both species are aggressive in nature and can grow from cut stems and seeds that fall on the soil during the flowering season and are able to sprout whenever the conditions become favourable. They colonise agricultural lands, abandoned sites, roadsides, railways, waterways and plantations (personal observation). In South Africa, *T. diversifolia* is invasive in the regions of Limpopo, Mpumalanga and along coastal regions of KwaZulu-Natal, while *T. rotundifolia* is invasive in the Gauteng, Mpumalanga, Limpopo and North West provinces [9]. Both species are viewed as environmental and ecological weeds in South Africa because of their allelopathic properties with the ability to compete with native vegetation for light and space [9, 10]. Due to their negative impacts, the two species have been declared category 1 weeds by The National Environment Management and Biodiversity Act [11]. Control measures such as chemical, mechanical and biological methods are applied to help reduce the spread of these weeds. Although investigations on biological control of these weeds using insects are currently in progress, some insects that feed on *T. diversifolia* do not feed on *T. rotundifolia*. Reasons for this are not known (personal communication with Simelane, ARC-PPRI Pretoria).

*Tithonia diversifolia* is exploited as a source of medicine in its native and most parts of its invasive regions to treat one or more ailment. The weed is used in Venezuela to treat abscesses [12]; in Mexico for malaria, hematomas and muscular pain [13]; in India for wounds and skin infections [14, 15]; in Taiwan for diabetes [16]; in Kenya for malaria and as an antidote for snake bite and to treat ectoparasites in cattle and to improve appetite [17]; in Uganda for microbial infection in sexual organs [18]; and in Nigeria for malaria [19]. The species is also used for poultry feed, as pesticides, compost for soil improvement and for bioremediation in Nigeria, Malawi, Kenya, Uganda and Zimbabwe [20–23]. Several workers have reported the extracts of the weed to possess antiviral, antidiabetic, anthelmintic, anti-inflammatory, antispasmodic, antiproliferation and antimalarial activities as well as analgesic properties [24–29]. Antibacterial and antifungal activity have also been investigated [30], however, there is no existing report on antimycobacterial activity. Phytochemicals such as phenolics, flavonoids, tannins, alkaloids, saponins and cardiac glycosides have been detected in the species [31]. A plethora of compounds which include sesquiterpenoids, diterpenoids and flavonoids have been isolated [32].

*Tithonia rotundifolia* has only been reported to be used as a source of medicine to treat fever in Venezuela [12]. Some compounds, including germacranolides, eudesmanolides and flavonoids have been isolated from this weed [32]. However, information on the biological activities of extracts and fractions and phytochemical quantification of this weed is scarce or non-existent. This study investigates and compares the antimicrobial activity of extracts and fractions of *T. diversifolia* and *T. rotundifolia* against selected pathogenic organisms and non-tuberculous mycobacteria, quantification of phytochemicals and identification of the bioactive compound(s) and toxicity levels.

## Methods

### Microbial strains

Microbial strains used in this study were *Escherichia coli* (ATCC 25922), *Enterococcus faecalis* (ATCC 29212), *Salmonella* Typhimurium (ATCC 700720), *Pseudomonas aeruginosa* (ATCC 27853), *Staphylococcus aureus* (ATCC 29213), *Salmonella* Dublin (ATCC 15480), *Mycobacterium smegmatis* (ATCC 1441), *M*. *aurum* (NCTC 10437), *M*. *fortuitum* (ATCC 6841) and BCG *M. bovis* Pasteur strain (P1172). *Klebsiella pneumoniae* was isolated from commercial chicken eggs [33]. *Aspergillus fumigatus* was isolated from a chicken with systemic mycosis, *Cryptococcus neoformans* from a cheetah and *Candida albicans* from a Gouldian finch.

### Plant collection and sample preparation

The leaves of *T. diversifolia* were collected from Umkomaas, south coast of KwaZulu-Natal, while the leaves of *T. rotundifolia* were collected from Pretoria North, Gauteng, in South Africa. Voucher specimens (Coll. 6 PRU 123728, *Tithonia diversifolia,* and Coll. 1 PRU 123725, *Tithonia rotundifolia*) were identified with help from the herbarium curator Mrs. Elsa van Wyk and deposited at the H.G.W.J. Schweickerdt Herbarium, University of Pretoria. Leaves of both plants were placed in the drying room of the Department of Paraclinical Sciences to dry, ground to powder and stored in sealed glass jars while preparing for experiments.

### Preparation of plant extracts and fractions for antimicrobial and toxicity assays

From each powdered sample, 4 g was weighed into centrifuge tubes, 40 ml of 70% ethanol (EtOH), hot distilled water, acetone, dichloromethane (DCM) and 50% methanol (MeOH) were added separately to each aliquot of powder. The mixtures were centrifuged at 300 x g for 10 min and filtered through Whatman No. 1 filter paper. The resultant extracts were transferred into pre-weighed labelled glass vials and the procedure was repeated twice on the marc to exhaustively extract plant material. Resultant extracts were placed under a stream of air to dry completely and stored in a dark room at 4 °C while preparing for the experiment. For fractionation 30 g of each plant material was extracted with absolute MeOH and allowed to dry. The dried extracts were fractionated with a separating funnel using hexane, chloroform, ethyl acetate and butanol. Various fractions were dried and used for the experiments.

### Extraction of plant material for phytochemical determination

Ten milligrams of 50% MeOH was added to 0.1 g dried plant samples weighed into a 50 ml centrifuge tube. The mixture was stirred using a spatula and centrifuged for 10 min at 300 x g. After centrifugation the mixture was filtered through Whatman No. 1 filter paper and the resultant extracts were used immediately to determine the phytochemicals in focus to prevent metabolite deterioration or decomposition.

### Antimicrobial screening

#### Culturing bacterial and fungal strains

Bacterial stocks were maintained on Mueller-Hinton (MH) agar while fungi were maintained on Sabouraud Dextrose (SD) agar. The agar were sterilized by autoclaving, poured into Petri dishes and allowed to gel. The plates were allowed to cool overnight and the stock bacterial and fungal strains were streaked and sub-cultured on the plates. The inoculated bacterial plates were incubated for 24 h at 37 °C while fungal plates were incubated for 48 h at 30 °C to allow the colonies to develop. *Mycobacterium* strains were cultured adopting the method of McGaw et al. [34] and maintained on Löwenstein-Jensen agar slants, supplemented with glycerol. Prepared cultures were stored at 4 °C for maintenance.

#### In vitro microdilution assay

The antibacterial, antifungal and antimycobacterial assays were carried out through the serial microdilution method in a 96-well microplates described by Eloff and Masoko et al. [35, 36]. To prepare the cultures, a single colony of each bacterial strain (*Klebsiella pneumoniae, Pseudomonas aeruginosa, Enterococcus faecalis*, *Escherichia coli*, *Staphylococcus aureus*, *Salmonella* Typhimurium and *S.* Dublin*)* was inoculated from agar plates into sterilized MH broth in sterile McCartney bottles incubated at 37 °C in an incubator with an orbital shaker for between 12 and 18 h. *Aspergillus fumigatus Cryptococcus neoformans* and *Candida albicans* cultures were prepared by inoculating each fungal species in sterilized SD broth in sterile McCartney bottles and incubating at 30 °C in an incubator with an orbital shaker for 24 h for *C. albicans* and 72 h for *C. neoformans* and *A. fumigatus*. Each culture was diluted in fresh MH/SD broth and the absorbance was measured at a wavelength of 560 nm using a microplate reader, compared to a McFarland standard No. 1, and used for the screening. To each well of a sterile 96-well microplate, 100 μl of sterile water was added followed by the resuspended plant extracts (10 mg/ml in 50% MeOH for the 50% MeOH extract, distilled water for hot water extract and acetone for the other organic solvent extracts and fractions) only on the first row. From the first row the mixture was serially diluted two-fold downwards (column 1–12: A to H). One hundred μl of the diluted culture were added to each well of the microplates afterwards. Similarly, 100 μl of 2 mg/ml gentamicin and Amphotericin B used as positive controls and 50% MeOH, distilled water, and acetone as negative controls were serially diluted. Also, a plate containing only sterile water and bacteria or fungi was also prepared as a guide for reading the minimum inhibitory concentrations (MIC). The microplates were incubated at 37 °C for bacteria and 30 °C for fungi for 24 h. To all wells of the incubated microplates, 40 μl of 0.2 mg/ml p-iodonitrotetrazolium chloride (INT) were added to the bacterial and 50 μl to the fungal plates. The last well with clear inhibition of bacterial or fungal growth as shown by a decrease in colour change of the INT was recorded as the MIC after 1 h of further incubation for bacteria and 24 and 48 h for fungi. The experiment was repeated twice with three replicates in each assay.

For the mycobacterial strains, cultures of *M*. *smegmatis*, *M*. *aurum*, *M*. *fortuitum* and *M. bovis* were prepared by mixing a few microbial colonies of each strain with sterile distilled water to attain a concentration of cells compared to a McFarland No. 1 solution. Cell suspensions were diluted with freshly prepared Middlebrook 7H9 broth supplemented with 10% oleic acid, albumin, dextrose, and catalase (OADC) to obtain a final inoculum density of approximately 4 × 10^7^ cfu/ml. Streptomycin and rifampicin were used as positive controls and sterile water, acetone and 50% MeOH as negative controls. The microdilution assay as described by Eloff [35] was applied to obtain the MICs.

### Phytochemical determination

#### Phytochemical detection

Phytochemicals were qualitatively detected in the plant extracts using standard procedures for phenolics, flavonoids and tannins [37–39], alkaloids [9, 40] and saponins [41].

#### Phytochemical quantification

Total phenolics, total flavonoids, flavonol and hydrolysable tannin contents were quantitatively determined following standard methods using freshly prepared 50% MeOH extracts. Following Folin-Ciocalteu (Folin-C) method [42] with some modifications total phenolic compositions of the plant extracts were evaluated. From each freshly prepared 50% MeOH plant extract 50 μl was transferred into test tubes (5 test tube replicates for each extract), 950 μl of sterile distilled water was added followed by the addition of 500 μl of 1 N Folin-C reagent and 2.5 ml of 2% sodium carbonate (NaCO_3_) in the dark. Different concentrations of gallic acid ranging from 0 to 150 mg/ml) were prepared and used as the standard. Also, a blank containing 50% MeOH in place of the plant extracts was prepared. The test tubes containing the mixtures were incubated at room temperature for 40 min, and 200 μl of the reacted mixtures were immediately transferred into 96-well plates and a microplate reader was used to measure the absorbance at 725 nm. Total phenolics were expressed as mg gallic acid equivalents (GAE) per gram dry weight.

The butanol-HCl assay as described by Makkar [42] was used to determine hydrolysable tannin content of the plant extracts. Five hundred microliters of plant extracts were aliquoted into test tubes and diluted to 10 ml with 50% MeOH. Three mg of butanol/HCl (95/5%) reagent was added followed by 100 μl of 2% ferric ammonium sulphate in 2 N HCl. The test tubes were loosely covered and heated for 50 min in a boiling water bath. After the test tubes were allowed to cool at room temperature the absorbance was read at 550 nm including an unheated mixture used as blank.

Adopting the method described by Abdel-Hameed et al. [43] with some modification, the total flavonoid content of the plant extracts was determined using the aluminium chloride. One hundred μl of plant extract was mixed with 100 μl of 20% aluminium chloride (AlCl_3_) and 2 drops of glacial acetic acid. The mixture was diluted to 3000 μl with 50% MeOH. Blank samples were prepared without AlCl_3_, and a standard curve was prepared using catechin (concentration between 0 and 150 mg/ml) in MeOH. Absorbance was read at 415 nm after 40 min. The total flavonoid content was expressed as mg catechin equivalent (CE) per gram dry plant material.

With some modification the aluminium chloride method described by Abdel-Hameed et al. [43] was used to quantify the flavonol content. One ml of 20 mg/ml of AlCl_3_ was added to 1 ml of each plant extract in a test tube followed by 3 ml of 50 mg/ml of sodium ethanoate (CH_3_COONa). A standard curve was prepared using catechin in MeOH. The mixtures were incubated for 2.5 h and absorbance was read at 440 nm. Flavonol content was expressed as mg catechin equivalent per gram of dry plant material.

#### UPLC/MS detection of active compound(s)

One milligram (1 mg/ml) of each fraction of both species was dissolved with 500 μl of acetonitrile and 500 μl water (UPLC grade) and sonicated for 5 min. From the mixture, 100 μl was pipetted into Eppendorf tubes and made up to 1 ml with acetonitrile and water and vortexed for 2 min. From the solution 100 μl was transferred into UPLC graded p-vials and made up to 1 ml. A blank containing acetonitrile and water was prepared and samples were loaded into the HPLC-HR-ESI-MS (Waters Acquity) Ultra Performance Liquid Chromatography (UPLC®) system hyphenated to a quadrupole-time-of-flight (QTOF) and were analyzed.

### Toxicological assays

#### In vitro cytotoxicity assay

The plant extracts were screened for cytotoxicity against Vero monkey kidney cells using the tetrazolium-based colorimetric (MTT) assay described by Mosmann [44]. The Vero cell line was chosen for the study due to its sensitivity to toxicity, easy to culture and availability and also following its recommendation as a model to detect basal cytotoxicity [45]. Vero cells were grown in Minimal Essential Medium (MEM) supplemented with 0.1% gentamicin (Virbac) and 5% foetal calf serum (Highveld Biological). Cells of a subconfluent culture were harvested and centrifuged for 5 min at 200 x g. followed by resuspension in MEM to attain a 5 × 10^4^ cells/ml. To each well of a sterile microplate 100 μl of cell suspension was pipetted into columns 2 to 11 and only MEM (200 μl) in columns 1 and 12 to maintain humidity. The plates were incubated at 37 °C in a 5% CO_2_ incubator for 24 h to allow the cells to attach and reach the exponential phase of growth. One hundred μl of the extracts at differing concentrations prepared in MEM was added to the plates in quadruplicate. The microtitre plates were further incubated for 48 h at 37 °C in a 5% CO_2_ incubator with the plant samples. Untreated cells, positive control (doxorubicin chloride, Pfizer Laboratories) and negative controls (acetone, 50% MeOH and distilled water) were also included. After incubation for 24 h, the MEM with plant extract was aspirated from the cells and washed with 150 μl phosphate buffered saline (PBS, Whitehead Scientific) and replaced with 200 μl of fresh MEM. Following the washing step, 30 μl MTT (Sigma, stock solution of 5 mg/ml prepared in PBS) was added to each well and the plates were incubated for a further 4 h at 37 °C. After incubation with MTT the medium and the MTT in each well were carefully removed, without disturbing the MTT crystals in the wells. The cells were washed with PBS and the MTT formazan crystals were dissolved by adding 50 μl DMSO to each well. The plates were shaken gently to allow the MTT solution to dissolve. The amount of MTT reduction was measured immediately by detecting absorbance in a microplate reader at a wavelength of 570 nm and a reference wavelength of 630 nm. The wells in columns 1 and 12, containing medium and MTT without cells, were used to blank the plate reader. The LC_50_ value was calculated as the concentration of plant samples resulting in a 50% reduction of absorbance compared to untreated cells.

#### In vitro genotoxicity assay

Genotoxicity of the plant samples was determined by testing the plant samples against *S*. *typhimurium* strains TA98 (for frameshift mutation) and TA100 (for base-pair mutation) using the plate incorporation assay (Ames test) as described by Maron and Ames [46]. Acetone, DCM and hot water extracts were dissolved initially in 10% DMSO and later diluted to the required concentrations (5, 0.5 and 0.05 mg/ml) under sterile conditions using sterile distilled water to reduce the DMSO concentration to 1%. The plant samples were filter-sterilized and each plant sample was tested against *S*. *typhimurium* strains TA98 and TA100 (100 μl/plate of a fresh overnight culture prepared by inoculating 100 μl stock bacteria in 10 ml Oxoid nutrient broth and incubating for 16 h at 37 °C) without exogenous metabolic activation. A positive control, 4-nitroquinoline-N-oxide (4-NQO) and negative controls 1% DMSO and sterilized water were also prepared. The plates were incubated for 48 h at 37 °C and colonies were counted manually using a colony counter.

### Statistical analysis

A Student t-test using GENSTAT statistical software, version 14.0 (VSN International Ltd., UK), was used to compare the amount of total phenolics, total flavonoids, flavonol content and hydrolysable tannins of the extracts. Where the test statistics were not significant, no post-hoc tests were done. Finally, a Student t-test was used to analyse cytotoxicity and genotoxicity data.

## Results

### Antimicrobial activity

#### Antibacterial activity

The solvent leaf extracts and fractions of *T. diversifolia* and *T. rotundifolia* exhibited different levels of inhibitory activity (from moderate to very good chosen with reference to Eloff [47] and Sánchez and Kouznetsov [48]) against all tested bacterial strains (Table 1). Ethanol (70%) and acetone extracts of *T. diversifolia* displayed good activity with MIC of 0.07 mg/ml against *E*. *faecalis*. The hexane and chloroform fractions were very active against *P. aeruginosa* (0.07 mg/ml). Hot water extract of *T. rotundifolia* showed very good activity against *E. coli* and *S*. Typhimurium with MIC of 0.01 mg/ml and good activity (0.07 mg/ml) against *E*. *faecalis*, followed by the acetone extract with MIC of 0.03 mg/ml against *E*. *faecalis*, 0.07 mg/ml against *K. pneumoniae*, *S. aureus* and *S*. Typhimurium. The DCM extract showed good activity (0.07 mg/ml) against *S. aureus.* Activities displayed by the different fractions of *T. rotundifolia* were moderate except for the butanol fraction which showed no inhibitory effect against all tested strains.

**Table 1:** Antibacterial activity of solvent extracts and fractions of *T. diversifolia* and *T. rotundifolia*

Plant species	Extract/fraction	MIC (mg/ml)						
		*Ec*	*Kp*	*Ef*	SD	*Pa*	*Sa*	ST
*T. diversifolia*	Ethanol	1.25	0.62	0.07*	0.31	1.25	0.31	0.62
	Methanol	0.62	0.62	0.62	0.62	1.25	0.62	**0.15**
	DCM	0.31	**0.15**	0.31	0.31	0.31	0.62	0.62
	Acetone	**0.15**	**0.15**	0.07*	0.31	0.31	0.62	0.31
	Hot water	1.25	0.62	2.5	1.25	1.25	**0.15**	0.62
	Hexane	1.25	0.31	0.62	0.62	0.07*	0.62	0.62
	Chloroform	1.25	0.62	0.31	1.25	0.07*	**0.15**	1.25
	Butanol	> 2.5	2.5	> 2.5	> 2.5	> 2.5	> 2.5	> 2.5
	ETAC	2.5	2.5	0.62	> 2.5	**0.15**	0.31	2.5
*T. rotundifolia*	Ethanol	0.31	**0.15**	0.31	0.62	0.62	0.07*	0.62
	Methanol	0.62	0.31	2.5	**0.15**	0.62	0.62	0.31
	DCM	0.62	**0.15**	**0.15**	**0.15**	0.31	0.07*	**0.15**
	Acetone	0.31	0.07*	0.03**	0.31	0.31	0.07*	0.07*
	Hot water	0.01**	0.62	0.07*	0.62	1.25	0.31	0.01**
	Hexane	2.5	0.62	1.25	1.25	0.62	1.25	1.25
	Chloroform	2.5	> 2.5	1.25	> 2.5	0.62	> 2.5	> 2.5
	Butanol	> 2.5	> 2.5	> 2.5	> 2.5	> 2.5	> 2.5	> 2.5
	ETAC	**0.15**	1.25	> 2.5	1.25	1.25	0.62	1.25
Gent 2 mg/ml (+ve con.)		0.016*	0.50	0.50	0.004**	0.008**	0.008**	0.08**

*DCM* dichloromethane, *ETAC* ethyl acetate fraction, *Gent* gentamicin, *+ve* positive control, *Ec Escherichia coli*, *Kp Klebsiella pneumonia*, *Ef Enterococcus faecalis*, *SD Salmonella* Dublin, *Pa Pseudomonas aeruginosa*, *Sa Staphylococcus aureus*, *ST Salmonella* Typhimurium, *T*. *diversifolia Tithonia diversifolia*, *T*. *rotundifolia Tithonia rotundifolia*

Values written in bold with no asterisk are considered moderate; Values written in bold with one asterisk are considered active; Values written in bold with two asterisks are considered very active; Values greater than 0.1 mg/ml but less than 2.5 mg/ml are considered as weak activity, while values greater than 2.5 are considered not active

#### Antimycobacterial activity

The MICs of antimycobacterial activity displayed by both species are presented in Table 2. Although most of the extracts and fractions of *T. diversifolia* displayed weak antimycobacterial activity, the DCM and acetone extracts were moderately active against *M*. *aurum* while only the acetone extract was moderately active against *M. bovis* BCG. Only the 70% EtOH and 50% MeOH extracts showed moderate activity against *M*. *smegmatis*. Among the extracts and fractions of *T. rotundifolia* tested, most of the activities ranged from weak to moderate. However, the DCM and acetone extracts showed good activity (0.07 mg/ml) against *M*. *aurum* and *M*. *smegmatis* and very good activity (0.03 mg/ml) against *M*. *fortuitum*. The acetone and ethyl acetate extracts inhibited the growth of *M. bovis* BCG at MIC 0.07 mg/ml, while the DCM extract had MIC of 0.03 mg/ml.

**Table 2:** Antimycobacterial activity of solvent extracts and fractions of *T. diversifolia* and *T. rotundifolia*

Plant species	Extract/fraction	MIC (mg/ml			
		*M. aurum*	*M. bovis*	*M. fortuitum*	*M. smegmatis*
*T*. *diversifolia*	Ethanol	0.62	0.31	**0.15**	**0.15**
	Methanol	0.31	0.62	0.31	**0.15**
	DCM	**0.15**	0.31	0.31	0.31
	Acetone	**0.15**	**0.15**	0.62	2.5
	Hot water	0.31	0.31	0.62	0.31
	Hexane	0.62	0.31	1.25	2.5
	Chloroform	0.62	0.31	> 2.5	> 2.5
	Butanol	0.62	2.5	2.5	2.5
	ETAC	0.62	0.62	0.31	2.5
*T*. *rotundifolia*	Ethanol	0.62	0.62	0.62	0.62
	Methanol	1.25	0.31	1.25	0.62
	DCM	0.07*	0.03**	0.03**	0.07*
	Acetone	0.07*	0.07*	0.03**	0.07*
	Hot water	> 2.5	> 2.5	2.5	1.25
	Hexane	**0.15**	**0.15**	0.62	0.31
	Chloroform	2.5	0.31	0.31	0.31
	Butanol	> 2.5	> 2.5	> 2.5	> 2.5
	ETAC	**0.15**	0.07*	**0.15**	**0.15**
Strep (+ve con)	–	> 0.01**	0.063	**> 1.0**	> 0.01
Rif (+ve con)	–	> 0.01**	> **1.0**	**0.063**	> 0.01

*DCM* dichloromethane, *ETAC* ethyl acetate fraction, *Strep* Streptomycin, *Rif* Rifampicin, *+ve* positive control, *M. aurum Mycobacterium aurum, M. bovis Mycobacterium bovis, M. fortuitum Mycobacterium fortuitum, M. smegmatis Mycobacterium smegmatis, T*. *diversifolia Tithonia diversifolia*, *T*. *rotundifolia Tithonia rotundifolia*

Values written in bold with no asterisk are considered as moderate; Values written in bold with one asterisk are considered active/good; Values written in bold with two asterisks are considered very active/very good; Values greater than 0.1 mg/ml but less than 2.5 mg/ml are considered as weak activity, while values greater than 2.5 are considered not active

#### Antifungal activity

Different levels of antifungal activity were displayed by the solvent extracts and fractions of both species at varying concentrations (Table 3). EtOH (70%), 50% MeOH and acetone extracts only showed good activity against *C. neoformans* at 48 h with MIC of 0.07 mg/ml in all cases. *Candida albicans* was only inhibited moderately by the acetone extract, while the hexane fraction was very active against *C. neoformans* at 48 h and good at 72 h (MIC 0.03 and 0.07 mg/ml) and had moderate activity (0.15 mg/ml) against *A. fumigatus* at 48 h and 72 h. The chloroform fraction was moderately active only against *C. neoformans* at 48 h. The DCM and acetone extracts of *T. rotundifolia* showed good inhibitory activity against *C. neoformans* (0.07 mg/ml) and moderate activity (0.15 mg/ml) at 72 h, and was active against *C. albicans* (0.07 mg/ml) only at 48 h. Only the acetone extract of *T. rotundifolia* showed good activity (0.07 mg/ml) against *A. fumigatus,* while the DCM extract was moderately active (0.15 mg/ml). The ethyl acetate fraction also showed moderate activity against *C. neoformans* and *C. albicans* at 48 h.

**Table 3:** Antifungal activity of solvent extracts and fractions of *T*. *diversifolia* and *T*. *rotundifolia*

Plant species	Extract/fraction	MIC (mg/ml)					
		*A. fumigatus*		*C. neoformans*		*C. albicans*	
		48 h	72 h	48 h	72 h	48 h	72 h
*T*. *diversifolia*	Ethanol	2.5	1.25	0.07*	2.5	2.5	2.5
	Methanol	0.62	0.62	0.07*	0.62	2.5	2.5
	DCM	0.62	1.25	**0.15**	0.31	2.5	2.5
	Acetone	**0.15**	0.31	0.07*	**0.15**	**0.15**	**0.62**
	Hot water	0.62	0.62	**0.15**	1.25	1.25	1.25
	Hexaane	**0.15**	**0.15**	0.03**	0.07*	1.25	1.25
	Chloroform	0.31	1.25	**0.15**	0.31	1.25	1.25
	ETAC	2.5	2.5	0.31	1.25	**0.15**	**0.15**
*T*. *rotundifolia*	Ethanol	1.25	1.25	**0.15**	0.62	1.25	1.25
	Methanol	0.62	1.25	0.31	0.62	2.5	2.5
	DCM	**0.15**	1.25	0.07*	**0.15**	0.07*	**0.15**
	Acetone	0.07*	1.25	0.07*	**0.15**	0.07*	**0.15**
	Hot water	2.5	> 2.5	0.62	2.5	> 2.5	> 2.5
	Hexane	0.31	1.25	0.62	1.25	**0.15**	**0.15**
	Chloroform	> 2.5	0.62	1.25	1.25	0.31	0.62
	Butanol	> 2.5	> 2.5	0.31	0.31	1.25	1.25
	ETAC	> 2.5	1.25	**0.15**	0.62	**0.15**	0.62
Amp B (+ve con)		0.03**	0.62	0.008**	0.125	0.03**	0.50

*DCM* dichloromethane, *ETAC* ethyl acetate fraction, *Amp* Amphotericin B, *+ve* positive control, *A. fumigatus Aspergillus fumigatus*, *C. neoformans Cryptococcus neoformans*; *C. albicans Candida albicans*

Values written in bold with no asterisk are considered as moderate; Values written in bold with one asterisk are considered active; Values written in bold with two asterisks are considered very active; Values greater than 0.1 mg/ml but less than 2.5 mg/ml are considered as weak activity, while values greater than 2.5 mg/ml are considered not active

#### Phytochemical determination

**Fig. 1:**
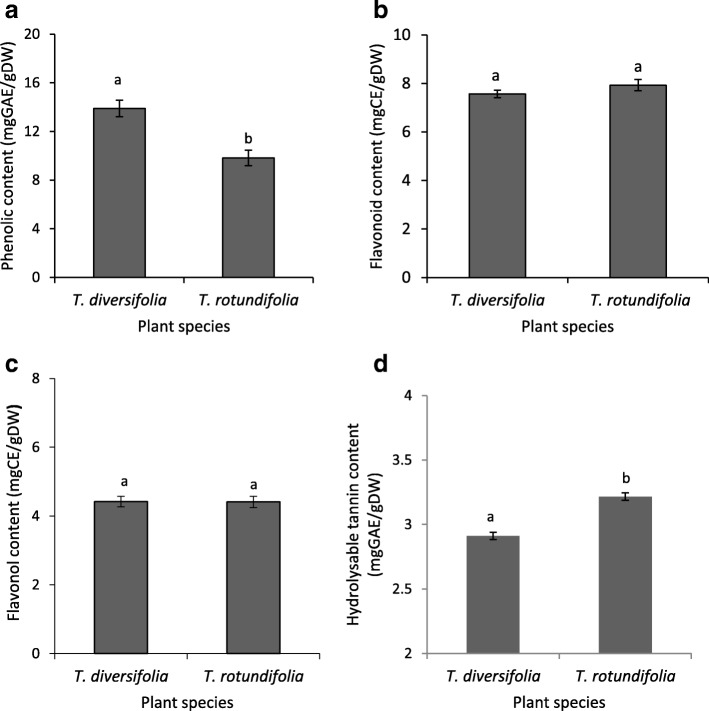
(**a**) Total phenolic content, as gallic acid equivalents, quantified in the leaves of *Tithonia diversifolia* and *T. rotundifolia* (**b**) total flavonoid content as catechin equivalents quantified in the leaves of *T. diversifolia* and *T. rotundifolia* (**c**) flavonol content as catechin equivalents quantified in the leaves of *T. diversifolia* and *T. rotundifolia* (**d**) hydrolysable tannin content as gallic acid equivalents quantified in the leaves of *T. diversifolia* and *T. rotundifolia*. Values in bars are means ±SEM. Sample sizes are given in parenthesis. DW = dry weight; GAE = gallic acid equivalents; CE = catechin equivalents

Results from this study showed that both species are rich in phenolics, flavonoids and tannins, while saponins were present in a moderate level, and low amounts of alkaloids were detected in both species (Table 4). Phytochemical quantification showed that *T. diversifolia* and *T. rotundifolia* are rich in phenolics, flavonoids and tannins (Fig. 1a-d). Phenolics (Fig. 1a) were higher in *T. diversifolia* with a significant difference (t_2_ = 4.34; *P* < 0.002). Although *T. rotundifolia* contained higher amounts of flavonoids (Fig. 1b) and flavonols (Fig. 1c) the differences were not statistically significant (t_2_, = − 1.29; *P* = 0.234) and (t_2_ = 1.14; *P* = 0.984). *Tithonia diversifolia* was higher in hydrolysable tannin content (Fig. 1d) with a statistical difference (t_2_ = − 2.92; *P* = 0.019).

**Table 4:** Phytochemicals qualitatively detected in leaf extracts of *T*. *diversifolia* and *T*. *rotundifolia*

Phyochemicals	*T*. *diversifolia*	*T*. *rotundifolia*
Phenolics	+++	+++
Flavonoids	+++	+++
Tannins	+++	+++
Alkaloids	+	+
Saponins	++	++

+ = present

++ = moderate

+++ = abundant

#### UPLC/MS analysis of active fractions

**Fig. 2:**
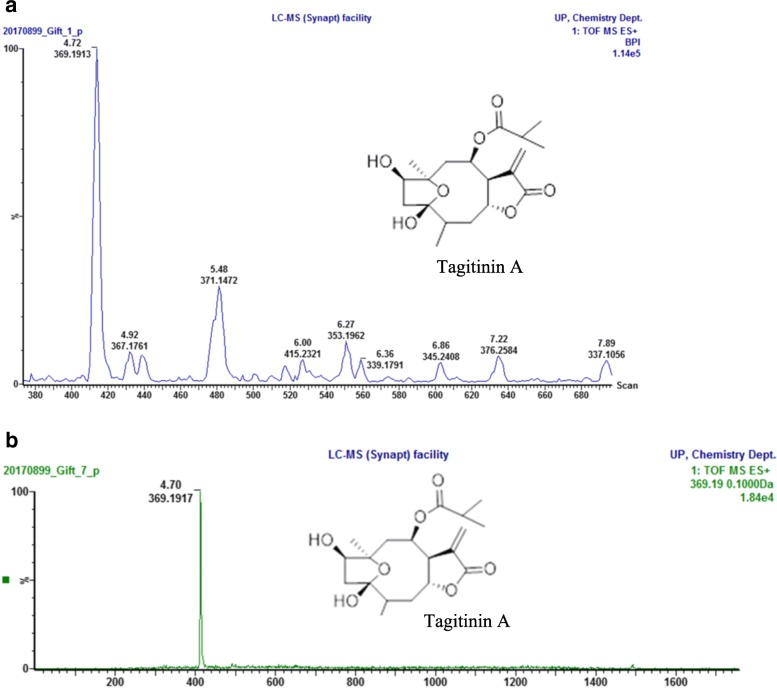
**a** TIC MS (Total ion chromatogram) of chloroform fraction of *T. diversifolia*. **b** Extracted-ion chromatogram (EIC) of tagitinin A from an ethyl acetate fraction of *T. rotundifolia*

UPLC-MS-ESI chromatogram of the active chloroform fraction of *T. diversifolia* appointed tagitinin A (molecular weight at 369.19 [M + H]^+^), as the major compound. In addition the Extracted-Ion Chromatogram (EIC) MS of the ethyl acetate fraction of *T. rotundifolia* showed a compound of molecular weight at 369.19 [M + H]^+^ which confirmed it as tagitinin A (Fig. 2).

### Toxicity assay

#### In vitro cytotoxicity

The results of the cytotoxicity test carried out on different solvent extracts of *T. diversifolia* and *T. rotundifolia* against Vero monkey kidney cells using the MTT colorimetric assay are presented in Table 5. The selectivity indexes for bacterial and fungal strains tested are also presented in Tables 6 and 7. In our study we refer to the lethal concentration (LC_50_) below 0.1 mg/ml to be toxic, and above 0.1 mg/ml to be less toxic or non-toxic depending on the range. Extracts with selectivity index (LC_50_ / MIC) above 10 are regarded as non-toxic, between 1 and 9 as less toxic and more active, and below 1 as toxic. Results showed that all extracts of *T. diversifolia* are toxic with LC_50_ values far lower than 0.1 mg/ml except the hot water extract with LC_50_ of 0.275 mg/ml. All extracts of *T. rotundifolia* tested were classified as being less toxic to non-toxic.

**Table 5:** LC_50_ values of *T*. *diversifolia* and *T*. *rotundifolia* solvent extracts against Vero Monkey kidney cells

Plant species	Extract	LC_50_ values (mg/ml)
*T. diversifolia*	Ethanol	0.004 ± 0.004
	Methanol	0.002 ± 0.001
	DCM	0.004 ± 0.001
	Acetone	0.012 ± 0.009
	Hot water	0.275 ± 0.04
*T. rotundifolia*	Ethanol	0.533 ± 0.113
	Methanol	0.518 ± 0.211
	DCM	0.938 ± 0.000
	Acetone	0.782 ± 0.089
	Hot water	0.841 ± 0.325
Doxorubicin +ve (μg/ml)		0.2009 ± 0.000

*DCM* dichloromethane

**Table 6:** Selectivity index of bacterial strains tested against *T*. *diversifolia* and *T*. *rotundifolia* solvent extracts

Plant species	Extract	Selectivity index (SI)						
		*Ec*	*Kp*	*Ef*	SD	*Pa*	*Sa*	ST
*T. diversifolia*	EtOH	0.0033	0.0065	0.0571	0.0129	0.0032	0.0129	0.0065
	MeOH	0.0032	0.0032	0.0032	0.0032	0.0016	0.0032	0.0133
	DCM	0.0129	0.0267	0.0129	0.0129	0.0129	0.0064	0.0065
	Acetone	0.0387	0.08	0.1714	0.0387	0.0387	0.0194	0.0387
	Hot water	0.220	0.4436	0.110	0.220	0.220	**1.8333**	0.4435
*T. rotundifolia*	EtOH	**1.719**	**3.553**	**1.719**	**0.861**	**0.861**	**7.614**	**0.861**
	MeOH	**0.836**	**1.671**	0.207	**3.453**	**0.836**	**0.836**	**1.671**
	DCM	**1.513**	**6.253**	**6.253**	**6.253**	**3.026**	13.4*	**6.253**
	Acetone	**2.523**	11.171*	26.067*	**2.523**	**2.523**	11.171*	11.171*
	Hot water	84.100*	**1.357**	12.014*	**1.357**	**0.673**	**2.713**	84.100*

*EtOH* ethanol, *MeOH* methanol, *DCM* dichloromethane, *Ec Escherichia coli*, *Kp Klebsiella pneumonia*, *Ef Enterococcus faecalis*, *SD Salmonella* Dublin, *Pa Pseudomonas aeruginosa*, *Sa Staphylococcus aureus*, *ST Salmonella* Typhimurium

Values written in bold with asterisk are non-toxic extracts with a safe margin and good promising antibacterial agent, while those written in bold with no asterisk are extracts with a relatively safe margin

**Table 7:** Selectivity index of fungal strains tested against *T*. *diversifolia* and *T*. *rotundifolia* solvent extracts

Plant species	Extract	Selectivity index (SI)					
		*A*. *fumigatus*		*C*. *neoformans*		*C*. *albicans*	
		48 h	72 h	48 h	72 h	48 h	72 h
*T. diversifolia*	EtOH	0.0016	0.0032	0.0571	0.0016	0.0016	0.0016
	MeOH	0.0032	0.0032	0.0286	0.0032	0.0008	0.0008
	DCM	0.0064	0.0032	0.0267	0.0129	0.0016	0.0016
	Acetone	0.080	0.0387	0.1714	0.08	0.08	0.0194
	Hot water	0.4436	0.4435	**1.8333**	0.22	0.22	0.22
*T. rotundifolia*	EtOH	0.426	0.426	**3.553**	**1.666**	0.426	0.426
	MeOH	**0.836**	0.414	**1.671**	**0.836**	0.207	0.207
	DCM	**6.253**	**0.750**	13.400*	**6.253**	13.400*	**6.253**
	Acetone	11.171*	**0.626**	11.171*	**5.213**	11.171*	**5.213**
	Hot water	0.336	NA	**1.357**	0.336	NA	NA

*EtOH* ethanol, *MeOH* methanol, *DCM* dichloromethane, *A*. *fumigatus Aspergillus fumigatus*, *C*. *neoformans Cryptococcus neoformans*, *C*. *albicans Candida albicans, NA* not available

Values written in bold with asterisk are non-toxic extracts on a safe margin and good promising antifungal agent, while those written in bold with no asterisk are extracts with a relatively safe margin

#### In vitro genotoxicity

Three (3) solvent extracts of *T. diversifolia* and *T. rotundifolia,* namely DCM, acetone and hot water were chosen for the genotoxicity assay. This is because, of the six (6) solvent extracts tested, these three showed a wide spectrum of antimicrobial activity. Results presented in Table 8 showed that none of the plant extracts was genotoxic or mutagenic against the tested strains, although this experiment was carried out without exogenous metabolic activation.

**Table 8:** Genotoxicity test of *T*. *diversifolia* and *T*. *rotundifolia* using *Salmonella typhimurium* strains TA98 and TA100 in the absence of exogenous metabolic activation

Plant species	Extracting solvent	Dose (μg/plate)	His + revertants plate	
			TA98	TA100
*T. diversifolia*	Acetone	5	27.33 ± 1.33	125.00 ± 1.2
		50	54.67 ± 1.20	116.33 ± 1.45
		500	45.00 ± 2.52	136.00 ± 2.0
	DCM	5	50.00 ± 1.15	104.33 ± 1.20
		50	51.33 ± 1.20	115.00 ± 2.00
		500	75.33 ± 0.33	117.3 ± 0.3
	Hot water	5	56.00 ± 3.00	117.00 ± 0.00
		50	62.00 ± 1.73	121.33 ± 0.58
		500	63.67 ± 2.52	132.33 ± 2.52
*T. rotundifolia*	Acetone	5	32.00 ± 1.53	122.00 ± 1.0
		50	25.00 ± 0.58	113.00 ± 1.15
		500	43.33 ± 1.67	108.67 ± 1.20
	DCM	5	35.33 ± 0.88	111.33 ± 1.76
		50	29.00 ± 2.08	119.00 ± 1.2
		500	26.67 ± 1.67	122.00 ± 1.7
	Hot water	5	45.00 ± 3.06	112.33 ± 4.04
		50	52.00 ± 3.00	112.00 ± 1.73
		500	59.00 ± 1.15	124.33 ± 3.06
4NQO + ve control			212.7 ± 1.9	538.7 ± 5.900
H_2_O –ve control			61.33 ± 1.45	130.00 ± 2.5
10% DMSO –ve control			57.00 ± 1.15	125.7 ± 1.9

*DCM* dichloromethane, *His* histidine, *−ve* negative control, *DMSO* dimethylsuphoxide, *H*_*2*_*O* water

## Discussion

*Tithonia diversifolia* and *T. rotundifolia* are known invasive weeds listed under category 1b of the National Environmental Management and Biodiversity Act [11] in South Africa, due to their negative impact on Agriculture, Ecology and Biodiversity. In a bid to control their spread, different approaches such as mechanical (clearing and digging out the roots), chemical (spraying with herbicides) and biological (use of insects to feed on the plants) control methods have been applied with low success rates. Reasons may be that the mechanical approach is labour intensive, chemical control may result in killing non-target species and, in using biological control which seems to be yielding a better result, there is a possibility that insects feeding on these plants may also be feeding on useful plants which are not the target (except for specialist insects). There is therefore a need to source a better and more sustainable way of controlling the spread of these plants. Regardless of the negative impact caused by these plants, their use in traditional medicine for the treatment of microbial related infections has also been documented [32]. Several studies have been carried out on the bioactivity, phytochemical screening and toxicity of *T. diversifolia*. However such studies on *T. rotundifolia* are scarce. It may not be impossible that good bioactivity possessed by *T. diversifolia* will also be present in *T. rotundifolia*, as several related compounds have been isolated from both species [32]. The current study did not only focus on comparing the antimicrobial activity of extracts and fractions of both species, but also investigated and identified important phytochemicals/compound(s) that may be responsible for such activity, and determined their toxicity levels.

### In vitro antimicrobial activity

#### Antibacterial/antimycobacterial activity of extracts and fractions of both species

From the results (Tables 1 and 2), the leaf extracts of *T. diversifolia* and *T. rotundifolia* showed a broad spectrum of antibacterial activity against the strains tested at varying concentrations. There was a selective activity of extracts and fractions effective against some strains and not effective against others. The growth of all bacterial strains tested were inhibited by *T. diversifolia* extracts and fractions (weak, moderate and good activities), except for the butanol fraction which did not inhibit the growth of *E. coli*, *E*. *faecalis*, *S*. Dublin, *P. aeruginosa*, *S. aureus* and *S.* Typhimurium. Our results agree with the findings of Obafemi et al. [30] for that of the methanolic extract against *E. coli* and *K. pneumoniae* though they applied the agar well diffusion method. These authors also reported the hexane extract to be effective against *E. coli* and *K. pneumoniae*, though we only tested the hexane fraction of this plant, which was also effective against these strains. Ethyl acetate fractions were also tested by Obafemi et al. [30] and were found to be effective against *E. coli*, *K. pneumoniae* and *Pseudomonas aeruginosa,* which supports our findings; however, we are reporting the activity displayed as weak. A sesquiterpene lactone isolated by these authors displayed MIC of 0.15 mg/ml for *E. coli*, *K. pneumoniae* and *P. aeruginosa* respectively. Linthoingambi and Singh [49] tested chloroform, petroleum ether and methanol extracts against *E*. *faecalis*, *S. aureus*, *P. aeruginosa* and *E. coli* and reported a weak activity of 6.25 mg/ml for the methanol extract for all strains tested, contrary to our results where we report MIC = 0.6 mg/ml for *E. coli*, *E*. *faecalis S. aureus* and 1.25 mg/ml for *P. aeruginosa* (though we regard this MIC as weak). Also from their findings the chloroform extract displayed MIC of 0.78 mg/ml against *E*. *faecalis* and *P. aeruginosa* while our findings showed MICs of 0.31 and 0.07 mg/ml for the chloroform fraction. The inhibitory activity of the chloroform fraction against *E. coli* (1.25 mg/ml) compared to their study was also referred to as weak in our study. John-Dewole and Oni [31] tested activity against *S. aureus*, *E. coli* and *P. aeruginosa* using water and methanol extracts but only the water extract showed some activity against *S. aureus* and *E. coli* at a high concentration of 12 mg/ml, while even at this concentration methanol showed no activity.

A study by Odeyemi et al. [50] also reported the inhibitory effect of the ethanol extract to *E. coli*, *P. aeruginosa* and *Enterococcus* and *Salmonella* species with MIC ranging from 1.25 to 5 mg/ml. Essential oils from *T. diversifolia* flowers have also been reported to inhibit the growth of *E. coli* and *K. pneumoniae* [51]. Inhibitory activity of *E. coli* by the 90% ethanol extract with a 10.4 mm zone of inhibition in the disc diffusion method was reported by Anthoney et al. [52]; however, the actual concentration at which the organism was inhibited was not clearly reported.

*Tithonia rotundifolia* extracts and fractions displayed a broad spectrum of antibacterial activity from weak to good activity against all tested strains. The hot water extract displayed very good inhibitory activity against *E. coli* and *S.* Typhimurium and good activity against *E*. *faecalis,* better than the positive control gentamicin used. This was followed by the acetone extract showing good activity against *K. pneumoniae*, *S. aureus* and *S*. Typhimurium and very good activity against *E*. *faecalis*. Comparing the antibacterial activity displayed by both species, it is clear that *T. rotundifolia* extracts exhibited overall best inhibitory effect against most of the tested strains, while the fractions of *T. diversifolia* displayed overall better activity compared to that of *T. rotundifolia*. Although the extracts and fractions of both species showed antimycobacterial activity, the activity displayed by *T. rotundifolia* extracts and fraction (s) was better than that of *T. diversifolia*. To the best of our knowledge, this is the first time the antimycobacterial activity of these species has been reported. However, the antimycobacterial activity of other plant species in the Asteraceae family has been reported [53].

#### Antifungal activity of extracts and fractions of *T. diversifolia* and *T. rotundifolia*

Antifungal activity was displayed by *T. diversifolia* and *T. rotundifolia* against *A. fumigatus*, *C. neoformans* and *C. albicans* at 48 and 72 h incubation. However, there was a differential growth response by the strains tested to different extracts and solvents of the species. At 48 h, ethanol, methanol and acetone extracts of *T. diversifolia* were very effective against *C. neoformans*, but there was a reduced activity at 72 h showing that the species was able to overcome the inhibitory effect to an extent. Only the hexane fraction was very effective against *C. neoformans* at 48 and 72 h. Activity displayed by other extracts and fractions against *A. fumigatus* and *C. albicans* was moderate to weak. Although information on the antifungal screening of *T. diversifolia* against the strains used in this study is scarce, an investigation carried out by Linthoingambi and Singh [49] in screening chloroform and methanol extracts of *T. diversifolia* against other fungal strains confirms the antifungal activity in this study. Agboola et al. [51] reported the essential oil from *T. diversifolia* to be effective against *Fusarium lateritum*, *F*. *solani* and *Cochliobolus lunatus*, though at a very high concentration of 72 mg/ml.

The DCM and acetone extracts of *T. rotundifolia* exhibited good activity against *C. neoformans* and *C. albicans* at MIC 0.07 mg/ml, but showed moderate activity at 72 h for both strains. Among all extracts tested only the acetone extract of *T. rotundifolia* showed good activity against *A. fumigatus* at 48 h. The fractions of *T. rotundifolia* were not as effective as the crude extracts. The results in Table 3 suggest that both species may be good antifungal agents that may be exploited as a source of antifungal treatment.

### Phytochemical analysis

#### Phytochemical detection and quantification

The phytochemicals identified in both species were phenolics, flavonoids, tannins, alkaloids and saponins. The flowers of *T. diversifolia* have been reported previously to contain phenolics, flavonoids, tannins and saponins [54, 55] but no alkaloids were detected. Though our results agree for phenolics, flavonoids, tannins and saponins, the absence of alkaloids in their investigation may be due to the plant part used. Our findings agree with those of John-Dewole and Oni [31] who also detected alkaloids in the leaves. Results from the phytochemical quantification (Fig. 1a-d) showed that both *T. diversifolia* and *T. rotundifolia* are rich in phenolics, flavonoids and hydrolysable tannins. Phenolics were higher in *T. diversifolia* and the difference was statistically significant (t_2_ = 4.34; *P* < 0.002). *Tithonia rotundifolia* contained higher amounts of flavonoid and flavonol contents but there were no significant differences (t_2_, = − 1.29; *P* = 0.234) and (t_2_ = 1.14; *P* = 0.984). Hydrolysable tannin content was higher in *T. rotundifolia* with a statistical difference (t_2_ = − 2.92; *P* = 0.019). The antimicrobial activity displayed by both species may be attributed to the major phytochemicals quantified in this study, as they are known to be responsible for other biological activities such as anti-inflammatory, antiviral, antioxidant and wound healing properties [56–58]. Compounds such as sesquiterpenes, diterpenes, and flavonoids have been isolated from *T. diversifolia* and *T. rotundifolia* [32]. Most of the compounds that have been isolated from both species are sesquiterpene lactones which are often present in the Asteraceae family [32, 59]. Most of the bioactivities reported in *T. diversifolia* have been attributed to sesquiterpene lactones such as tagitinin C, 1β-methoxydiversifolin and tagitinin A and chlorogenic acids [6, 60]. The antimicrobial activity displayed in this study may also be due to the presence of these compounds. The LC-MS-ESI chromatogram of the active chloroform fraction of *T. diversifolia* delivered a major compound with molecular weight at 369.19 [M + H]^+^, which was identified as tagitinin A followed by tagitinin C. In addition, the EIC-MS of the ethyl acetate fractions of *T. rotundifolia* also showed tagitinin A (Fig. 2) as the major constituent. From our results we can assume that tagitinin A may be the major antimicrobial agent responsible for activity in both species especially in *T. rotundifolia* which displayed the overall best antimicrobial activity. However, antimicrobial evaluation of this compound from the plant species is paramount to ascertain this fact.

#### In vitro toxicity test

##### Cytotoxicity test

The cytotoxicity screening of plants helps to determine the safety level at which products made from such plants may be consumed that is not harmful. From our screening, we observed that *T. diversifolia* was toxic for all the extracts tested except for the hot water extract with LC_50_ values greater than 1 mg/ml (Table 5) at concentrations between 0.012 to 1 mg/ml. The selectivity index values for both bacterial and fungal strains were less than 1 mg/ml except for the hot water extract against *S. aureus* and *C. neoformans* which showed a moderate activity. Some extracts of *T. rotundifolia* were less toxic while others were not (LC_50_ ranging from 0.518 to 0.938 mg/ml) and had selectivity index values above 1 except for the methanol extract which showed weak activity against *E*. *faecalis* and *C. albicans*. The antibacterial and antifungal activities displayed by *T. diversifolia* may be due to the toxicity of the plant extracts which may have influenced their activity against the strains tested. Although we have not come across any in vitro study of *T. diversifolia*, in vivo studies have shown that the plant is toxic. Elufioye [60] tested the 70% ethanol extract of the aerial parts of *T. diversifolia* in an in vivo study on Wistar rats at doses of 400, 800 and 1600 mg/kg. Between 30 min and 24 h of exposure, the extract showed both haematological and acute toxic effects on the kidney and liver. In an in vivo study carried out by Fakunle and Abatan [61], haematological changes were observed in rats administered an aqueous extract of *T. diversifolia* at doses between 100 and 200 mg/kg. Another study carried out by Adebayo et al. [62] on the aqueous extract at doses of 100 and 200 mg/kg administered to Wistar rats for 7 days showed that at 100 mg/kg the extract was relatively safe but at 200 mg/kg the extract was toxic. Aqueous extracts administered to Wistar rats at 10 mg/kg and 100 mg/kg for 90 days were reported to be relatively safe [63]. Some of the compounds reported to be responsible for toxicity of this plant are sesquiterpene lactones and chlorogenic acids [63, 64]. The presence of sequiterpene lactones in *T. diversifolia* may lead to kidney damage while the chlorogenic acids may result in liver injuries if the plant is not used with caution [63]. Though there are many sesquiterpenes present in *T. diversifolia* the most studied are the tagitinins which have been attributed to its pharmacological activity and these tagitinins have also been isolated from *T. rotundifolia* [32]. Although there are different classes of tagitinins such as A, B, C, D and E present in both species, tagitinin C has been discovered to be the main compound [65]. A further study has shown that though both species contain tagininin C, there are two other derivatives of tagitinin C that are only present in *T. diversifolia,* namely tagitinin C 2-methylbutyrate and 1β, 2α –epoxytagitinin C [32, 65, 66]. The toxicity levels displayed by *T. diversifolia* may be connected to the presence of these two compounds; however further screening is needed to ascertain this.

##### Genotoxicity test

According to the Ames test, a sample is considered to be mutagenic or genotoxic if the revertant colonies of the test sample double the revertant colonies of the negative control(s), or if there is a dose dependent increase in the number of colonies with the sample [67]. The genotoxicity tested carried out on the DCM, acetone and hot water extracts of *T. diversifolia* and *T. rotundifolia* against TA98 and TA100 of *Salmonella* strains using the Ames test did not show any genotoxic effect. Although, *T. diversifolia* was toxic as shown in our cytotoxicity result (Table 5), it was not mutagenic in the Ames test. There is a need for continuous screening of this plant to ascertain if it has a selective toxicity which may be helpful in the treatment of cancer cells as related studies have been carried out by some researchers [68–70].

## Conclusions

The problem caused by alien invasive plants cannot be over emphasized as they result in loss of agricultural lands, livelihoods and biodiversity which may lead to economic burden. Results from our study have shown that weeds such as *T. diversifolia* and *T. rotundifolia* may serve as a good source of antimicrobial drugs. Other work has been done on the antibacterial and antifungal activities, phytochemistry and toxicity levels of *T. diversifolia*. However, this study is the first to: (i) evaluate the antibacterial, antimycobacterial and antifungal activities of *T. rotundifolia*, (ii) determine the phytochemicals and identify the major compound that may be responsible for such activity, and (iii) determine the cytotoxicity and genotoxicity of different extracts of the plant. This is also the first study to compare the antimicrobial activity, quantify important phytochemicals such as phenolics, flavonoids and hydrolysable tannins in both *T. diversifolia* and *T. rotundifolia,* and also the cytotoxicity and genotoxicity levels of the different solvent extracts. The UPLC/MS analysis identified tagitinin A as a major constituent of active fractions of both plants. Our study is the first to screen these plants for genotoxicity. Though both species showed good antimicrobial activity, *T. rotundifolia* displayed a broader spectrum of antimicrobial activity. The low toxicity level displayed by *T. rotundifolia* extracts qualifies the plant for more biological screening of the extracts and compounds and also to determine the mechanism of action. This may lead to the production of novel antimicrobial drugs from this plant that may be used for the treatment of microbial infection and other opportunistic pathogenic infections in humans and animals. The economic burden caused by both species may be tackled through their use as a source of medicines. The weed plants may also serve as alternatives to highly exploited indigenous plants that possess the same medicinal properties. However, care must be taken in the use of *T. diversifolia* as it may be toxic.

## Abbreviations

4-NQO: 4-nitroquinoline-N-oxide
AlCl_3_: Aluminium chloride
butanol-HCl: Butanol-hydrochloride
CE: Catechin equivalents
CH_3_COONa: Sodium ethanoate
CO_2_: Carbon dioxide
DMSO: Dimethylsuphoxide
GAE: Gallic acid equivalents
HCl: Hydrochloric acid
HPLC-HR-ESI-MS: High Performance Liquid Chromatography- High Resolution-Electrospray Ionization-Mass Spectrometry
INT: p-iodonitrotetrazolium chloride
LC_50_: Lethal concentration
MEM: Minimal Essential Medium
MIC: Minimum inhibitory concentration
NaCO_3_: Sodium carbonate
OADC: oleic acid, albumin, dextrose, and catalase
PBS: Phosphate buffer saline
QTOF: Quadrupole-time-of-flight
UPLC: Ultra Performance Liquid Chromatography
